# The ethics of data mining in healthcare: challenges, frameworks, and future directions

**DOI:** 10.1186/s13040-025-00461-w

**Published:** 2025-07-11

**Authors:** Mohamed Mustaf Ahmed, Olalekan John Okesanya, Majd Oweidat, Zhinya Kawa Othman, Shuaibu Saidu Musa, Don Eliseo Lucero-Prisno III

**Affiliations:** 1 Faculty of Medicine and Health Sciences, SIMAD University, Mogadishu, Somalia; 2 Department of Public Health and Maritime Transport, University of Thessaly, Volos, Greece; 3 Department of Medical Laboratory Science, Chrisland University, Ajebo, Abeokuta, Nigeria; 4 College of Medicine, Hebron University, Hebron, Palestine; 5 Department of Pharmacy, Kurdistan Technical Institute, Sulaymaniyah, Kurdistan Region Iraq; 6 School of Global Health, Faculty of Medicine, Chulalongkorn University, Bangkok, Thailand; 7 Department of Nursing Science, Ahmadu Bello University, Zaria, Nigeria; 8 Department of Global Health and Development, London School of Hygiene and Tropical Medicine, London, UK; 9 Center for Research and Development, Cebu Normal University, Cebu, Philippines; 10 Center for University Research, University of Makati, Makati City, Philippines

**Keywords:** Data mining, Healthcare ethics, Privacy, Algorithmic bias, Data security, Patient consent

## Abstract

Data mining in healthcare offers transformative insights yet surfaces multilayered ethical and governance challenges that extend beyond privacy alone. Privacy and consent concerns remain paramount when handling sensitive medical data, particularly as healthcare organizations increasingly share patient information with large digital platforms. The risks of data breaches and unauthorized access are stark: 725 reportable incidents in 2023 alone exposed more than 133 million patient records, and hacking-related breaches surged by 239% since 2018. Algorithmic bias further threatens equity; models trained on historically prejudiced data can reinforce health disparities across protected groups. Therefore, transparency must span three levels–dataset documentation, model interpretability, and post-deployment audit logging–to make algorithmic reasoning and failures traceable. Security vulnerabilities in the Internet of Medical Things (IoMT) and cloud-based health platforms amplify these risks, while corporate data-sharing deals complicate questions of data ownership and patient autonomy. A comprehensive response requires (i) dataset-level artifacts such as “datasheets,” (ii) model-cards that disclose fairness metrics, and (iii) continuous logging of predictions and LIME/SHAP explanations for independent audits. Technical safeguards must blend differential privacy (with empirically validated noise budgets), homomorphic encryption for high-value queries, and federated learning to maintain the locality of raw data. Governance frameworks must also mandate routine bias and robust audits and harmonized penalties for non-compliance. Regular reassessments, thorough documentation, and active engagement with clinicians, patients, and regulators are critical to accountability. This paper synthesizes current evidence, from a 2019 European re-identification study demonstrating 99.98% uniqueness with 15 quasi-identifiers to recent clinical audits that trimmed false-negative rates via threshold recalibration, and proposes an integrated set of fairness, privacy, and security controls aligned with SPIRIT-AI, CONSORT-AI, and emerging PROBAST-AI guidelines. Implementing these solutions will help healthcare systems harness the benefits of data mining while safeguarding patient rights and sustaining public trust.

## Introduction

Healthcare professionals and researchers use data mining, which is a sophisticated analytical technique for extracting valuable insights and patterns to analyze large datasets, including electronic health records, medical imaging data, and data from wearable devices. Data mining enables the extraction of meaningful information by navigating extensive data collection, allowing the healthcare sector to uncover valuable knowledge across various applications [1, 2]. This technique has become increasingly important in many areas of life, including business, healthcare, social media, and government [2]. As data mining has become increasingly popular, important ethical questions have been raised [3]. These questions revolve around issues such as privacy and informed consent, ensuring that individuals authorize the use of their data for research. Additionally, fairness and equity in data usage must be considered to avoid potential biases and ensure ethical practices [4]. Data mining techniques have found significant applications in the healthcare sector, presenting vast opportunities to enhance patient treatment, optimize operational processes, and facilitate medical discoveries. The medical field has emerged as a key domain for leveraging these analytical methods, offering substantial benefits across various aspects of healthcare delivery and research [1]. Data mining helps to predict disease outbreaks, identify high-risk patients, personalize treatment plans, and improve overall patient outcomes [5]. For instance, by analyzing patient data, healthcare providers can predict which individuals are more likely to develop certain conditions, allowing early intervention and preventive care [5]. Data mining also plays a significant role in drug discovery, helping researchers identify potential new treatments by analyzing large molecular and genetic datasets [6]. Classification algorithms are employed to categorize patients into risk groups or to diagnose diseases based on symptoms and test results [7]. Clustering techniques help identify patterns in patient populations, which can be useful for targeted interventions or resource allocation [8]. Association rule mining is also used to discover the relationships between different medical conditions or treatments, potentially uncovering new insights into disease progression and treatment effectiveness [9].

The significance of data mining in healthcare is immense, with the potential to revolutionize healthcare delivery by making it more efficient, personalized, and effective. By leveraging the power of data, healthcare providers can make more informed decisions, researchers can accelerate scientific discoveries, and patients can receive better care [9]. However, the use of data mining in healthcare raises several ethical concerns. Confidential and private handling of health-related information is crucial because of its highly sensitive nature [3]. Patients may not always be aware of how their data are used in research. Additionally, there are concerns regarding the potential for bias in data mining algorithms, which could lead to unfair or discriminatory healthcare practices [10]. Recent research underlines the scale and urgency of these ethical concerns. In 2023 alone, 725 reportable breaches exposed more than 133 million patient records in the United States, an all-time high that represents a 239% increase in hacking incidents since 2018 [11]. Comparable upward trends are reported across Europe and Asia, Europe experienced a 35% year-over-yar increase in weekly cyber-attacks in Q2 2024, reaching about 1 367 attacks per organization per week [12]. APAC (Asia-Pacific) saw 2 510 attacks per organization weekly during the same period [12]. Systematic reviews published in 2024–2025 also highlight the inadequacy of “consent-by-default” models, calling for fine-grained dynamic consent and stronger oversight of secondary data use [13–15]. At the same time, privacy-enhancing technologies are rapidly evolving; state-of-the-art surveys show that differential privacy can preserve model utility at modest noise budgets, whereas homomorphic encryption and federated learning remain cost-prohibitive for routine clinical deployment [16–18]. Finally, new comparative analyses of data-mining ethics across healthcare, education and government sectors emphasise the need for cross-domain governance frameworks that blend technical safeguards with enforceable accountability mechanisms [19, 20]. As the healthcare industry continues to embrace data mining, it is important to address these ethical challenges to ensure that the benefits of this technology are realized while protecting patient rights and maintaining public trust. This paper examines the ethical dimensions of data mining in healthcare, using the industry as a critical example of the benefits and challenges of this technology. Healthcare is an area where data mining can do a lot of good, such as helping doctors make better decisions and find new treatments for diseases. However, it is also an area where people’s information is extremely sensitive and personal. We explored the main ethical problems that arise from the use of data mining in healthcare. These include concerns about patient privacy, ensuring that data are used fairly, and keeping information safe from misuse.

### Ethical issues in data mining

Data mining in healthcare presents significant ethical challenges that must be carefully addressed to ensure that patient rights are protected while harnessing the benefits of this technology [21]. These ethical issues primarily revolve around privacy and consent, algorithmic bias, transparency, accountability, and security (Table 1), as discussed below.

**Table 1:** Ethical issues in healthcare data mining

Ethical Issue	Description	Source(s)
Privacy & Consent	Risk of exposing private information without patient knowledge or consent. Patients may not be fully aware of how their data is used or shared. Anonymization techniques may not be sufficient to protect patient privacy.	[21–23]
Algorithmic Bias	Algorithms can perpetuate biases based on sensitive attributes like race or gender, leading to unfair outcomes in healthcare decisions. Historical data reflecting societal biases can be preserved in algorithms.	[24, 25]
Transparency & Accountability	Numerous computational processes function as “black boxes”, making it challenging to comprehend their decision-making processes and outcomes. Lack of transparency can be concerning medical decisions impacting patients’ lives.	[26, 27]
Security Concerns	Healthcare data is a valuable target for cybercriminals, leading to data breaches and severe consequences for patients. Insider threats and IoMT devices pose additional security challenges.	[28–32]

### Privacy and consent

One of the most pressing ethical issues in healthcare data mining is the protection of individual privacy [32]. Healthcare data are highly sensitive and contain personal information about patients’ medical conditions, treatments, and genetic makeup [21, 32]. When these data are mined, there is a risk of exposing private information without the patient’s knowledge or consent. The question of consent is particularly complex in healthcare data mining. Patients may not be fully aware of how their data are used or shared [23]. While many healthcare providers obtain general consent for data use, the specifics of data mining applications may not be communicated [23]. This raises ethical questions regarding the need for informed consent and patient autonomy issues. Anonymization techniques are commonly used to protect privacy during data mining processes. These techniques aim to make it difficult to trace information back to individual patients and allow insights without exposing personal identities [33]. However, the effectiveness of these techniques has been increasingly questioned. With the large amount of available data and advanced data mining techniques, it is becoming easier to re-identify individuals from supposedly anonymized datasets [34]. This challenges the notion that anonymization alone is sufficient to protect patient privacy. This limitation suggests that relying solely on anonymization may not adequately protect individual privacy.

### Algorithmic bias

A major ethical issue in healthcare data mining is the possible presence of bias in algorithms [24]. Data mining algorithms can inadvertently introduce or perpetuate biases, particularly when dealing with sensitive attributes, such as race, gender, or socioeconomic status [24]. This can lead to unfair or discriminatory outcomes in healthcare decisions and resource allocation. For instance, a data mining algorithm trained on historical datasets reflecting societal prejudices may replicate these biases in their output, including forecasts and recommendations [25]. This could result in certain groups receiving suboptimal care or being unfairly targeted for intervention. Addressing algorithmic bias requires careful consideration of the data used to train the algorithms and the implementation of fairness measures during the data mining process.

### Transparency and accountability

Achieving transparency and accountability in healthcare data mining models presents significant challenges despite their importance. The inner workings of many data mining algorithms, especially those using sophisticated machine learning methods, are often obscure, making it challenging to comprehend their decision-making processes [26, 27]. In the medical field, the lack of transparency in decision-making processes can raise concerns, as these choices may have significant consequences for patients’ health. Nevertheless, enhancing the interpretability and explainability of these models remains difficult [27]. Healthcare providers and patients must understand how decisions are made to trust and effectively use insights generated by data mining. Additionally, clear accountability structures are needed to determine responsibility when data mining leads to adverse outcomes.

### Security concerns

With the emergence of the Internet of Things (IoT), our tangible world is developing a new digital dimension [20]. Services, applications, and platforms associated with the Internet of Medical Things (IoMT) employ a common architectural framework. In this structure, data are collected by wearable devices or other medical equipment and subsequently transmitted to cloud storage [28]. The storage and processing of these cumbersome healthcare datasets for data mining purposes raises significant security concerns. This is because healthcare data are a valuable target for cybercriminals, and data breaches can have severe consequences for patients, including identity theft and discrimination [29].

Recent studies have highlighted the growing threat of insider attacks in the healthcare industry [30, 31]. These insider threats can compromise patient data and undermine the integrity of data mining efforts [31]. Protecting against these threats requires strong security measures and careful data-access management. Moreover, as healthcare increasingly adopts IoMT technology, new security challenges have emerged. Therefore, the large amounts of data generated by IoMT devices present both opportunities for data mining and risks for data breaches [32]. Thus, balancing the potential benefits of data mining with robust security measures remains an ongoing ethical challenge.

### Scenarios highlighting ethical challenges in healthcare data mining and privacy

Concerns about patient privacy have greatly increased because of the convergence of technology and healthcare, especially in data mining. Hospitals and healthcare networks are increasingly providing patient data to large digital businesses, such as Amazon, which raises several ethical concerns [35]. The possibility of third parties, particularly hackers, gaining illegal access to private patient data is one of the main problems. Patients frequently lose control over their data when data-gathering organizations are acquired by larger corporations. This ownership transfer may lead to new businesses using patient data without the required authorization, which raises serious ethical questions regarding patient autonomy and data rights [36]. Amazon’s healthcare initiative, demonstrated by Amazon Comprehend Medical, aims to address the difficulties associated with excessively large patient datasets. This tool helps pharmaceutical companies, hospitals, and researchers make sense of large medical datasets. However, Comprehend Medical is not fully compliant with health insurance portability and accountability act (HIPAA) regulations, even though Amazon asserts that it complies with certain requirements for managing protected health information (PHI) [37].

The PillPack–ReMy Health dispute illustrates how data-sharing intermediaries can expose prescription histories to unvetted third parties; Surescripts revoked ReMy Health’s access in 2019 after alleging fraudulent taps on its e-prescribing network [38], Concerns regarding the ethical implications of sharing genetic data have also been raised by personal genomics companies, such as 23andMe. These businesses help people find their lineage and other personal information, but they also frequently provide research organizations and pharmaceutical corporations with access to anonymized genetic data [39, 40]. The increasing availability of data on individuals’ genetic composition, reactions to medications, multi-omics responses, and genomic profiles is gradually steering healthcare towards tailored treatment approaches [41]. Therefore, genetic data can be used to intentionally cause harm. Recently, advances in genomics and precision medicine have provided scientists with the ability to tailor medical treatments to an individual’s genetic makeup or profile. When misused, this information can be used to design a toxin or poison that specifically targets a person’s genetic vulnerability and causes harm to them. Additionally, this practice raises significant ethical concerns about informed consent and the absence of monetary remuneration for individuals whose data are used in drug development and scientific research.

While people may donate their data for research out of benevolence, the monetization of such data may result in situations where the advances made possible by the contributions do not benefit the persons themselves [40]. The issue of patient privacy is further complicated by the potential for re-identifying anonymized data breaches. Seminal work at Carnegie Mellon University showed that ZIP code, birth date and sex uniquely identify 87% of U.S. residents [42]. A study showed that 15 demographic variables uniquely identified 99.98% of U. residents in a de-identified census dataset [34]. These findings underscore the persistent re-identification risk in ostensibly anonymised datasets, even when healthcare organisations apply safeguards such as k-anonymity, l-diversity and differential-privacy masking [44], or enforce dynamic-consent workflows, legally binding data-use agreements and continuous audit-trail logging [45]. The security and integrity of patient data remain seriously threatened in the absence of these combined technical and governance controls. The 2015 Anthem breach alone exposed the records of more than 80 million health-insurance members, underscoring systemic vulnerability [46]. This trend underscores the fragility of healthcare information systems and emphasizes the urgent need for enhanced security. Medical institutions, including hospitals, walk-in clinics, pharmacies, and health insurance providers, possess highly valuable data, making them attractive targets for cybercriminals to steal data. This situation is exacerbated by the relatively weak security infrastructure prevalent in the healthcare industry, further increasing the likelihood of successful attacks.

 A report by Security Scorecard ranked healthcare 9th in terms of overall security ratings among industries, highlighting its susceptibility to breaches [ 47, 48 ]. Healthcare data breaches have widespread consequences, affecting 26% of consumers in the United States. Half of these affected individuals experience medical identity theft, resulting in an average personal expense of $ 2, 500. In August 2016, two significant breaches occurred in the dam. NewKirk Products, a company that issues healthcare ID cards, suffered a breach that impacted 3.47 million patients [ 49 ]. During the same month, Banner Health faced a security incident that not only compromised patient records but also affected their payment system data [ 48 ]. Medical Informatics Engineering was breached in July 2015, affecting 3.5 million patients and exposing sensitive data, including social security numbers and diagnoses [ 49 ]. Advocate Health Care faced a major breach in August 2013 that exposed the personal and medical information of 4 million patients due to unencrypted records being compromised during theft [ 50 ]. Community Health Systems experienced a breach affecting 4.5 million patients between April and June 2014 [ 47 ]. Among significant data breaches, the TRICARE incident in September 2011 exposed the information of 4.9 million military members and their dependents. Additionally, in September 2015, the Excellus BlueCross BlueShield breach compromised sensitive personal and health data belonging to more than 10 million subscribers [ 48 ].

**Fig. 1:**
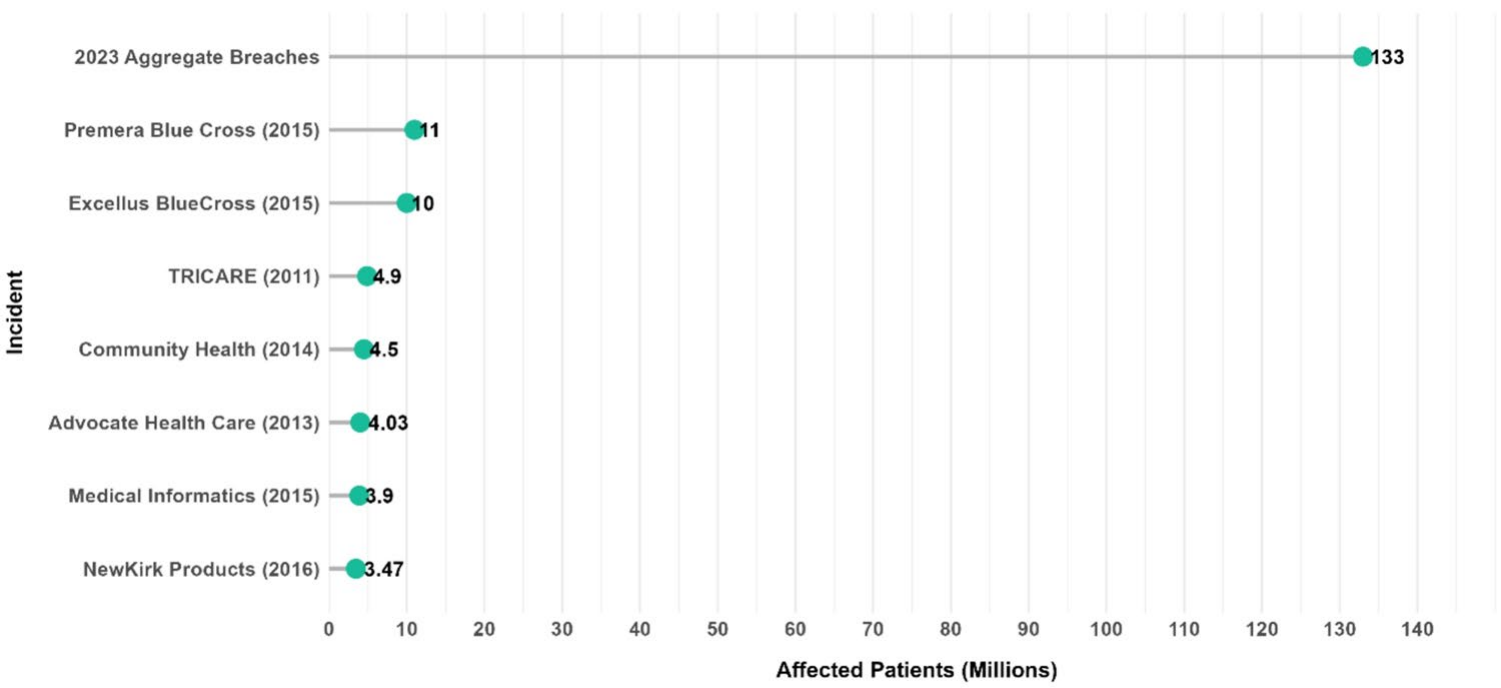
Major healthcare data breaches [11, 46, 47]

Premera Blue Cross announced a breach affecting more than 11 million people, with hackers gaining access to Social Security numbers and bank account details [47, 48]. Healthcare data breaches have increased in size and frequency over the past 14 years, raising ethical concerns. In 2023, 725 breaches exposed over 133 million patient records, with hacking and ransomware attacks accounting for nearly 80% of the incidents (Fig. 1). These breaches often expose sensitive personal and health-related information, exacerbating medical identity theft and causing financial harm. The shift from physical to digital records is intended to enhance healthcare efficiency and increase data vulnerability. Technological advancements, such as data encryption, reduced data loss, and theft, occurred between 2009 and 2015. However, hacking has emerged as the predominant cause of breaches, with a 239% increase from 2018 to 2023.

The severity of these breaches raises ethical issues regarding patient privacy, data security, and the healthcare industry’s responsibility to protect sensitive information. The increasing frequency and severity of breaches demand stronger cybersecurity protocols and a higher emphasis on ethical data governance, especially in the era of data-driven healthcare advancements [11]. Ethical concerns regarding the use of patient data are becoming increasingly prominent as technology businesses join healthcare organizations more frequently. In the era of healthcare data mining, concerns about the possible misuse of patient information resulting from corporate mergers, fraudulent activities, and illegal access highlight the critical need for extensive ethical frameworks and strict rules to safeguard patient privacy and confidentiality. However, the security and integrity of patient data are seriously threatened in the absence of such protections [34, 37].

### Ethical implications of data mining for global healthcare systems

Data mining in global healthcare systems presents several ethical challenges that must be addressed to prevent exacerbating breaches, misuse of data, and existing disparities, and to ensure equitable outcomes across diverse populations. These challenges include quantifying the impact of data mining processes, which can perpetuate biases, and model generalizability, which can lead to over- or under-treatment in specific populations. Regular audits of data-driven models for bias and their impact on clinical outcomes are essential, particularly in regions with pronounced socioeconomic and racial disparities [51, 52]. Model generalizability is another significant ethical challenge, as models trained on data from one region or hospital may perform poorly when deployed in another region with different demographics or resources. This issue is critical, as models may be applied in regions with different disease prevalence, healthcare infrastructure, and population characteristics, leading to diagnostic inaccuracies and suboptimal care for underrepresented or marginalized populations [53].

Transparency in model and data documentation is vital for ethical decision making in global healthcare, as poorly documented models and datasets can obscure biases or data collection flaws, leading to unintended consequences in clinical practice. Comprehensive documentation, such as detailed data sheets for datasets, can reveal how data are collected and highlight any sources of discrimination that are inherent in the model. Co-developing documentation tools, such as model cards, with healthcare practitioners helps formalize processes and ensures that ethical considerations, such as potential bias and trade-offs, are addressed before model deployment [54, 55]. The regulation of data mining models for healthcare is currently underdeveloped, raising significant ethical concerns, especially in global settings (Table 2). Although some regulations are in place, they do not adequately address the unique challenges presented by machine learning models in healthcare across various contexts. Extensive regulatory frameworks are needed to evaluate the safety and efficacy of these models, consider health inequalities during their development and implementation, and incorporate provisions for health equity assessments. Additionally, these frameworks should consider the legal ramifications of using ML in healthcare, including issues related to malpractice and liability [54, 56].

Several frameworks now touch directly on AI in health; however, none provide end-to-end protection against misuse. The EU AI Act (Regulation (EU) 2024/1689) classifies clinical decision-support tools as high-risk and therefore imposes mandatory risk management, transparency reports, and post-market monitoring [57]. The Act, however, leaves model-bias auditing and dataset provenance checks to future secondary legislation, with enforcement at member-state level that may be uneven [57]. In the United States, HIPAA safeguards the confidentiality of electronic health information but is silent on algorithmic bias, model drift, and explainability duties [58]. Draft FDA guidance released in January 2025 proposes a life-cycle approach for “AI-enabled Device Software Functions”, yet it is limited to devices seeking market clearance and does not cover hospital-built algorithms or retrospective research models [59]. The General Data Protection Regulation (GDPR) grants patients a right “not to be subject to a decision based solely on automated processing” [60]. However, GDPR focuses on personal data protection and does not compel developers to publish bias metrics or allow external auditing of clinical AI. Moreover, the regulation does not harmonize health-data retention rules across member states, complicating cross-border model validation [60]. Global bodies are also responding to this. WHO’s 2024 guidance on large multi-modal models lists forty recommendations covering governance, procurement, and equity, yet remains non-binding [61]. Likewise, the US Sect. 1557 Final Rule on Nondiscrimination in Health Programs prohibits biased clinical decision-support tools but offers no technical standard for measuring fairness [62]. These examples show that current regulations (a) address privacy without bias and transparency, (b) address life-cycle quality without open auditing, or (c) are aspirational and unenforceable. Therefore, a comprehensive governance strategy requires routine bias-and-robustness audits, mandatory publication of data lineage, real-time incident reporting, and harmonized penalties for non-compliance that extend beyond financial fines to the suspension of algorithm use.

**Table 2:** Ethical implications of data mining for global healthcare systems

Ethical Challenge	Description	Source(s)
Perpetuation of Biases	Data mining processes can amplify existing disparities, leading to inequitable outcomes across diverse populations.	[52, 53]
Model Generalizability	Models trained on data from one region may perform poorly in regions with different demographics or resources, leading to diagnostic inaccuracies and suboptimal care for underrepresented groups; this is critical in regions with differing disease prevalence, healthcare infrastructure, and population characteristics.	[54]
Transparency & Documentation	Poorly documented models and datasets can obscure biases or data collection flaws, leading to unintended consequences in clinical practice.	[55, 56]
Regulation Underdevelopment	Current regulations often fail to address the specific challenges posed by healthcare machine learning models in diverse global settings.	[55, 57]

### Ethical solutions and frameworks for responsible healthcare data mining

**Fig. 2:**
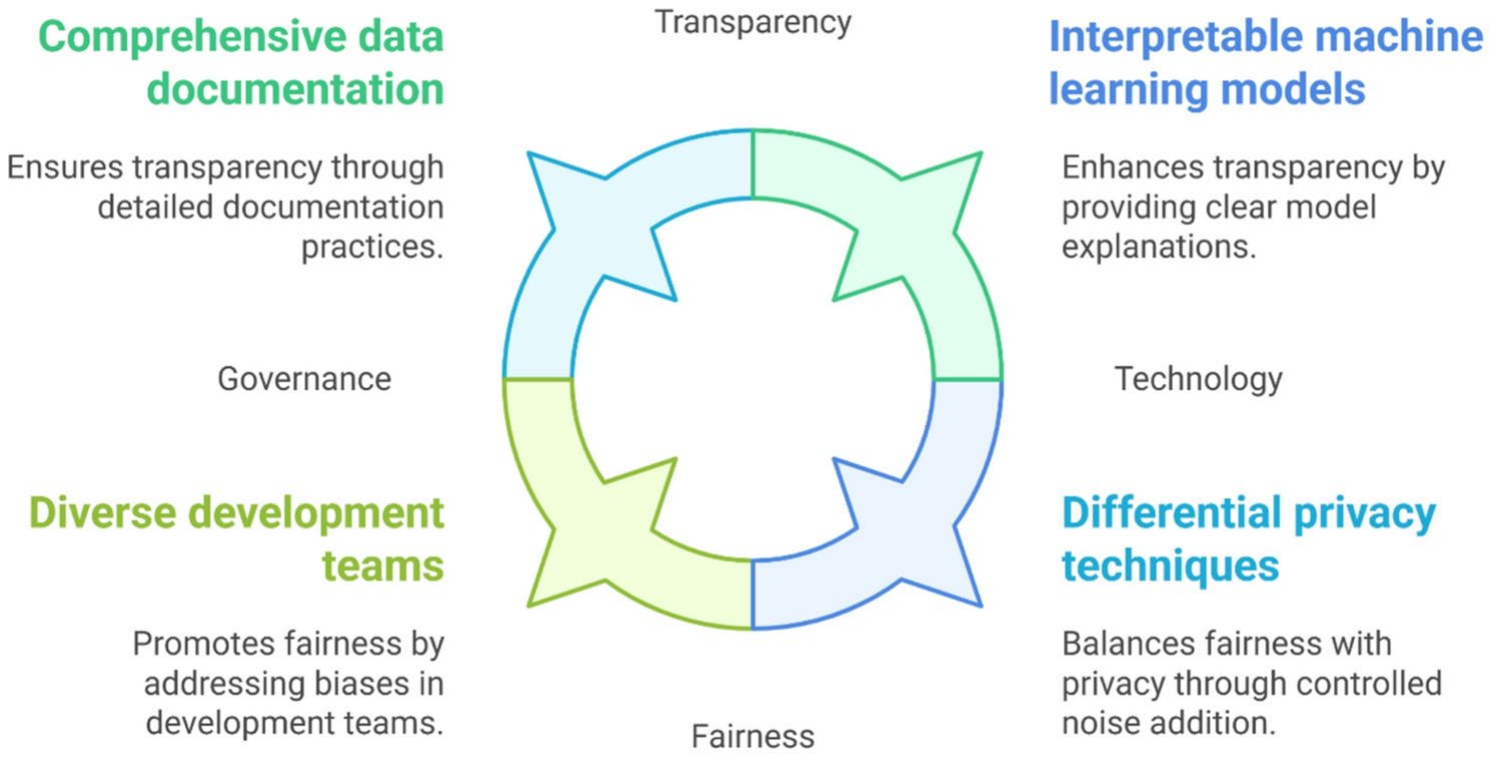
Ethical frameworks for responsible healthcare data mining

Several solutions and ethical frameworks can be implemented to address the ethical concerns associated with data mining in healthcare (Fig. 2). These approaches focus on data governance, algorithm fairness, privacy-enhancing technologies, and transparency.

### Data governance

The implementation of strong data governance protocols is essential for ethical healthcare data-mining practices. A thorough data governance structure should provide explicit directives for the acquisition, maintenance, application and distribution of data [63]. The structure should contain the creation of comprehensive consent documents that explicitly outline the intended use of patient information in data-mining processes [64]. These forms should be written in plain language and be easily understandable by patients. The framework should also focus on implementing policies to collect and retain only the data necessary for specific healthcare purposes, thereby reducing the risk of unnecessary data exposures [65]. It is essential to implement rigorous security measures to limit access to confidential healthcare information and ensure that only authorized individuals can view or handle these data [66]. This includes the implementation of role-based access control systems and regular audits of data access logs [66]. Additionally, the framework should involve creating policies for the proper handling of data throughout its lifecycle, including guidelines for data retention and secure data destruction when it is no longer needed [67].

### Fairness in algorithms

In clinical prediction, fairness is most often operationalised as equal opportunity, which requires identical sensitivity across protected groups, or equalised odds, which requires identical sensitivity and specificity [68, 69]. Other metrics include demographic parity and predictive parity [70]. Selecting one metric over another is a policy choice because the trade-offs differ between high-risk decision contexts (e.g., critical-care triage) and screening or case-finding applications [69, 71]. Large audit studies continue to show clinically meaningful bias. A study found that fewer than a quarter of the 20 included studies reported any fairness metric and most failed to analyze performance across demographic subgroups [72]. Comparable disparities have been documented for radiology triage tools, sepsis alerts and hospital readmission models, underscoring the need for systematic mitigation [73–75]. Fairness interventions can be applied before, during, or after training. *Pre-processing* techniques, such as stratified sampling, re-weighting and synthetic oversampling with methods like SMOTE-IPF, seek to balance under-represented groups prior to learning [76]. *In-processing* approaches introduce adversarial debiasing or fairness-constrained loss functions, including equalised-odds regularization, to reduce disparate error rates while training is in progress [77]. *Post-processing* methods adjust decision thresholds or perform calibration-by-group so that false-positive and false-negative rates converge without retraining the base model [78]. Finally, *life-cycle monitoring* recognizes that fairness drifts over time; therefore, metrics must be logged continuously and a re-audit triggered whenever model inputs, population mix, or performance metrics change in clinically significant ways [79]. Several guidelines now embed explicit fairness requirements, each specifying the minimum amount of information that researchers and developers must disclose (Table 3).

**Table 3:** Fairness requirements in key AI-health reporting guidelines

Guideline	Year*	Fairness-specific requirement	Source(s)
SPIRIT-AI	2020	Trial protocols must describe plans for bias testing across demographic sub-groups during prospective evaluation.	[81]
CONSORT-AI	2020	Published trial results must be reported separately for each protected sub-group so differential performance can be assessed.	[82]
STARD-AI	2021 (protocol)	Diagnostic-accuracy studies must present performance metrics stratified by sex, ethnicity, age, or other key demographics.	[83]
TRIPOD + AI	2024	Prediction-model papers must state which fairness metrics were calculated, describe any mitigation steps, and show external validation across demographic strata.	[84]
PROBAST + AI	2025	The new Bias & Equity domain asks seven signaling questions on data representativeness, subgroup performance, and monitoring for fairness drift.	[85]

Note: * Years refer to the official publication date of each guideline or its protocol

These frameworks collectively recommend that developers report model performance across demographic strata, document any mitigation techniques employed, and make source codes or audit notebooks publicly accessible so that external groups can replicate fairness analyses. A pragmatic workflow begins by defining the fairness metric in collaboration with clinical and organizational stakeholders; equal opportunity is often preferred for diagnostic applications, whereas equalised odds is suited to high-stakes triage [68, 85]. A baseline audit is then conducted on retrospective data; if the difference in sensitivity across any protected group exceeds 5% points, mitigation is required [86]. The chosen pre-, in- or post-processing technique is implemented, and model performance is re-evaluated to confirm that disparities have narrowed without unacceptable loss of overall accuracy [86]. The final model is registered in an institutional AI registry with documentation that satisfies SPIRIT-AI and CONSORT-AI requirements [80, 81]. Continuous monitoring should follow, with a scheduled re-audit every six months or whenever demographic drift exceeds 10%, ensuring that fairness remains stable over time. This structured approach translates abstract ethical principles into concrete actions aligned with the emerging international consensus on responsible AI in health care.

### Privacy-enhancing technologies

 Protecting patient information requires more than a catalogue of advanced privacy-enhancing technologies; it also means recognizing their current limitations and integrating them with robust, system-level security controls [ 87 ]. Differential privacy, for example, guarantees formal protection only by injecting statistical noise; recent evaluations on clinical datasets show that common ε-values (≤ 1) reduce model AUROC by up to 7% points and erase minority-group signal, undermining downstream equity analyses [ 16, 72 ]. Recent benchmarks indicate that fully-homomorphic inference on medical images can still take 20–180 min per case depending on model depth, while encrypted pipelines may be 30 × slower than plaintext baselines even after GPU acceleration [ 88, 89 ]. Federated learning mitigates data-residency barriers but does not prevent model inversion or gradient-leakage attacks; clinical pilots report communication overheads that increase training time by a factor of eight in multi-hospital settings [ 90, 91 ]. Equally important, privacy breaches often stem from basic cybersecurity lapses rather than from analytic workflows. Healthcare remains the world’s most-targeted critical-infrastructure sector, recording 386 publicly reported cyber-attacks in the first ten months of 2024 alone, a trajectory that threatens to eclipse the 2023 peak [ 92 ]. Ransomware operators now demand average payouts exceeding US $1.6 million, and highly publicized breaches such as the 2024 Medibank incident in Australia demonstrate how exfiltrated medical records can be weaponized for extortion and identity theft [ 93, 94 ]. Accordingly, privacy-preserving analytics must sit within a layered defense that includes a zero-trust network architecture, encryption-at-rest, vulnerability patch management, and continuous intrusion detection. Only by combining technical privacy mechanisms with foundational security practices can health systems reduce both accidental disclosure and malicious compromises.

### Transparency

Transparency in healthcare AI is commonly addressed at three complementary levels, dataset documentation, model interpretability, and post-deployment audit logging [95–97]. Recent multi-center studies illustrate how local-explanation techniques can both uncover clinically relevant signals and expose hidden failure modes. Several multi-center audits have used SHAP to reveal sex-specific and vital-sign-specific biases in sepsis-triage algorithms [98, 99]. In cardiac-risk prediction, investigators applied LIME-style local explanations to digitized 12-lead ECGs, allowing cardiologists to confirm that QRS-complex morphology, rather than incidental demographic features, drove high-risk alerts; the explanations showed strong qualitative agreement with electrophysiologist annotations [100]. A study paired Grad-CAM with SHAP to show that transformer-based model LungMaxViT attended to clinically relevant lung fields; retraining on balanced data improved AUC from 0.926 to 0.932 over the MaxViT baseline [101]. Local methods such as LIME build a sparse linear surrogate around each prediction; the resulting coefficients form a ranked list of feature contributions in the original clinical units, for example, showing how a higher serum-lactate value increases a patient’s predicted risk [102, 103]. When these attribution vectors are stored in audit logs, quality-assurance teams can trace outlier decisions back to their root causes, thereby satisfying transparency and accountability mandates [104]. However, interpretability alone is insufficient; model cards, version control, external-validation reports, and timestamped prediction logs complete the transparency stack and must accompany any visual explanation.

## Conclusion

The ethical challenges surrounding data mining in healthcare demand immediate attention, as technology continues to revolutionize medical care and research. The dramatic rise in breaches, 725 incidents, and more than 133 million records compromised in 2023, coupled with a 239% increase in hacking events since 2018, prove that today’s ad hoc safeguards are no longer adequate. Convergence with Big Tech ecosystems, large-language-model integrations, and Internet of Medical Things (IoMT) devices now exposes patient data across a vastly expanded attack surface. Addressing this landscape requires a layered strategy that integrates (i) dataset-level artifacts (datasheets) to document provenance, (ii) model-level disclosures (model cards) that publicize fairness and robustness metrics, and (iii) post-deployment audit trails capturing prediction logs and explanation vectors. Advanced privacy technologies, differential privacy calibrated with empirically validated noise budgets, homomorphic encryption for high-value queries, and federated learning to keep raw data local, must be paired with routine bias-and-robustness audits, dynamic consent mechanisms, and harmonized penalties that can suspend unsafe algorithms. Cultivating a culture of transparency and accountability within healthcare organizations is as critical as the technology stack. This means adopting governance frameworks mapped to SPIRIT-AI, CONSORT-AI, TRIPOD + AI, and the forthcoming PROBAST-AI Bias & Equity domain, ensuring that every stage, design, deployment, and monitoring meets scrutinized ethical benchmarks. Policymakers must enforce cross-border data-retention standards, while technology companies and providers must collaborate on secure, interoperable infrastructures. Ultimately, the future of healthcare data mining hinges on systems that are simultaneously powerful, fair, and verifiably safe. Achieving this vision will require the sustained, coordinated effort of clinicians, data scientists, cybersecurity experts, ethicists, regulators, and patients themselves. Only through such multi-stakeholder collaboration can we unlock the life-saving potential of data-driven medicine without eroding the public trust on which healthcare depends.

## Data Availability

No datasets were generated or analysed during the current study.
